# Indoleamine 2,3-Dioxygenase (IDO) Activity: A Perspective Biomarker for Laboratory Determination in Tumor Immunotherapy

**DOI:** 10.3390/biomedicines11071988

**Published:** 2023-07-13

**Authors:** Pengbo Yang, Junhua Zhang

**Affiliations:** 1 Department of Laboratory Medicine, Beijing Hospital, National Center of Gerontology, Institute of Geriatric Medicine, Chinese Academy of Medical Sciences, Beijing 100730, China; 2 The Key Laboratory of Geriatrics, Beijing Institute of Geriatrics, Institute of Geriatric Medicine, Chinese Academy of Medical Sciences, Beijing Hospital/National Center of Gerontology of National Health Commission, Beijing 100730, China

**Keywords:** indoleamine 2,3-dioxygenase, kynurenine pathway, immune response, biomarker, enzymatic activity, IDO inhibitors

## Abstract

Indoleamine 2,3-dioxygenase 1 (IDO1) is a heme enzyme involved in catalyzing the conversion of tryptophan (Trp) into kynurenine (Kyn) at the first rate-limiting step in the kynurenine pathway of L-tryptophan metabolism. It has been found to be involved in several biological functions such as aging, immune microorganism, neurodegenerative and infectious diseases, and cancer. IDO1 plays an important role in immune tolerance by depleting tryptophan in the tumor microenvironment and inhibiting the proliferation of effector T cells, which makes it an important emerging biomarker for cancer immunotherapy. Therefore, the research and development of IDO1 inhibitors are of great importance for tumor therapy. Of interest, IDO activity assays are of great value in the screening and evaluation of inhibitors. Herein, we mainly review the biological functions of IDO1, immune regulation, key signaling molecules in the response pathway, and the development of IDO1 inhibitors in clinical trials. Furthermore, this review provides a comprehensive overview and, in particular, a discussion of currently available IDO activity assays for use in the evaluation of IDO inhibitors in human blood. We believe that the IDO activity is a promising biomarker for the immune escape and laboratory evaluation of tumor immunotherapy.

## 1. Introduction

**Figure 1:**
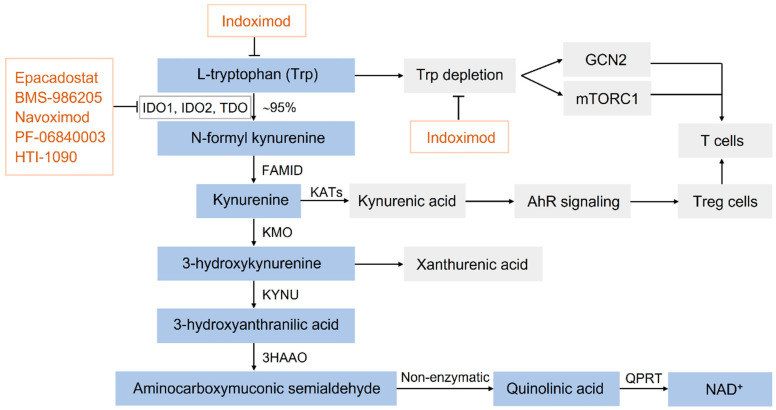
Metabolism of tryptophan and the action of IDO (indoleamine 2,3-dioxygenase) inhibitors in the tryptophan metabolic pathway. In vivo, ~95% of L-tryptophan is catabolized via IDO in the form of kynurenine degradation products. Additional enzymatic metabolites in the pathway promote immune suppression. IDO inhibitors primarily compete with tryptophan by binding to the catalytic domain of IDO or non-competitive mechanisms to affect tryptophan metabolism, thereby exerting an inhibitory effect on IDO. IDO1, indoleamine 2,3-dioxygenase 1; IDO2, indoleamine 2,3-dioxygenase 2; TDO, tryptophan dioxygenase; FAMID, formamidase; KATs, kynurenine aminotransferases; KMO, kynurenine 3-monooxygenase; KYNU, kynureninase; 3HAAO, 3-hydroxyanthranilic acid dioxygenase; QPRT, quinolinic-acid phosphoribosyl transferase; NAD^+^, nicotinamide adenine dinucleotide; GCN2, general control nondepressible-2; mTORC1, mammalian target of rapamycin complex 1; AhR, aryl hydrocarbon receptor; Treg cells, regulatory T cells.

L-Tryptophan (Trp) is the least abundant essential amino acid with an indole ring backbone, which is required for a variety of cellular activities and normal physiological functions [1]. Trp is metabolized via four major pathways: decarboxylation for producing tryptamine, protein synthesis, the serotonin pathway, and the kynurenine (Kyn) pathway (KP), where ~95% of Trp is degraded through the KP as the substrate for indoleamine-2,3-dioxygenase 1 (IDO1), generating N-Formyl kynurenine (NFK) [2]. NFK is rapidly converted to Kyn, catalyzed via formamidase (FAMID). Kyn can then be metabolized to kynurenic acid through the activation of kynurenine aminotransferases (KATs), participating in the aryl hydrocarbon receptor (AhR) signaling pathway. Meanwhile, kynurenine 3-monooxygenase (KMO) catalyzes Kyn to 3-hydroxykynurenine, which is subsequently converted to 3-hydroxyanthranilic acid by Kynureninase (KYNU). And then 3-hydroxyanthranilic acid is metabolized to quinolinic acid (QUIN). QUIN has toxic effects but are metabolized to the de novo nicotinamide adenine dinucleotide (NAD^+^) biosynthesis pathway [3,4] (Figure 1).

In addition to the IDO, KATs, and KMO described above, the kynurenine pathway involves other important enzymes, such as tryptophan dioxygenase (TDO) and 3-hydroxyanthranilic acid dioxygenase (3HAAO) [5,6]. In mammals, the three rate-limiting catabolic enzymes, i.e., IDO1, IDO2, and TDO2, can break the indole ring, each of which has specialized tissue-specific and regulated expression [7,8,9]. They play an important role in regulating immune response and tumor immune escape. Although IDO1, IDO2, and TDO2 have similar biochemical properties, they are different in structure, substrate conversion rate, substrate specificity, tissue distribution, and expression regulation [9,10,11]. IDO1 can be expressed in many types of biological tissues, and it increases with age in specific tissues [12,13]. IDO2 is a genetic paralog of IDO1 and is directly adjacent to IDO1 on the same chromosome. The expression level of IDO2 is low in a more restricted set of tissues, including liver, kidney, lymph nodes, and placenta [14]. Similarly, the expression of TDO2 is relatively small, predominantly in the liver where it plays a key role in maintaining the systemic homeostasis of the tryptophan levels [15]. Here, we will focus on the biology of IDO1 because, of the three enzymes, it has the highest capacity to catalyze the Trp metabolism, while simultaneously being the tryptophan-metabolizing enzyme most closely linked to immune function [16].

IDO works along the tryptophan-depleting kynurenine pathway and might trigger an immunosuppressive microenvironment in tumors, which in turn allows tumor cells to escape immune recognition and cytotoxicity [17]. Therefore, the IDO activity involved in KP plays an important role in the laboratory diagnosis of multiple types of human cancers. An interesting study performed by Botticelli et al. shows that higher kynurenine/tryptophan (Kyn/Trp) ratio representing IDO1 activity could predict resistance to anti-PD-1 treatment in NSCLC, which suggest the possibility of using anti-PD-1 plus IDO inhibitors in those patients with a high level of Kyn/Trp ratio. Consequently, IDO activity has been suggested as a possible mechanism of resistance to anti-PD-1 treatment, leading to an immunosuppressive microenvironment [18,19]. Recently, it was also reported that platinum-resistant lung tumors utilized both IDO1 and TDO2 enzymes for survival in order to escape immune surveillance [20]. In conclusion, high IDO activity can serve as a prognostic and predictive biomarker to optimize the immunotherapy of patients with lung tumors [21]. In this review, we summarized the characteristics of IDO as a biomarker in clinical trials, focusing on various methods to determine IDO activity so far.

## 2. Immune Regulation of IDO Pathway

**Figure 2:**
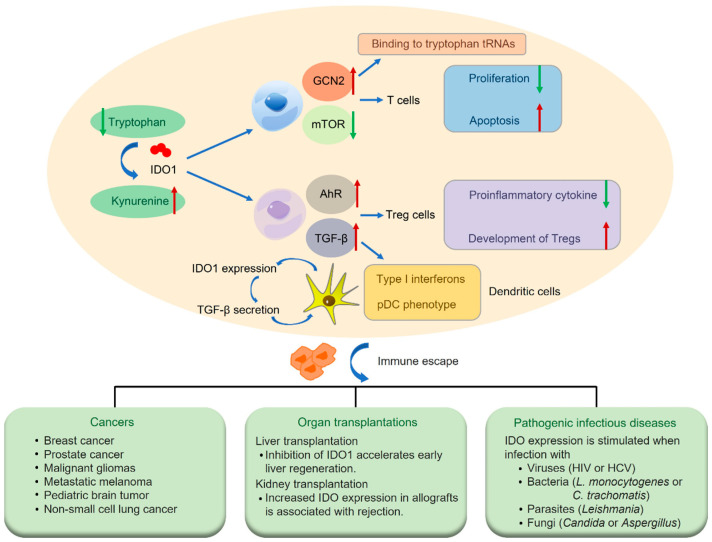
Regulatory effect of IDO on T cells and Tregs in immune microenvironment. IDO depletes tryptophan and exerts important immunosuppressive functions via the regulation of T effector cells and the induction of T regulatory cell proliferation. This immune response eventually results in clinical conditions, such as cancer, infection, and transplantation. Tregs, regulatory T cells; GCN2, general control nondepressible-2; mTOR, mammalian target of rapamycin; AhR, aryl hydrocarbon receptor; TGF-β, tumor growth factor β. Red arrow, an increase; green arrow, a decrease.

IDO1 (EC 1.13.11.42), a heme-containing dioxygenase, assumes a key role in catalyzing the catabolism of mammalian L-tryptophan [22,23]. Studies have confirmed that human IDO1 is a monomeric enzyme with high enzymatic activity for the conversion of Trp to Kyn and its metabolites [24]. IDO pathways have important effects on T cells in response to antigenic stimulation (Figure 2). It activates the tryptophan metabolic pathway, causing a large depletion of Trp. T cells stagnate in G1 phase due to severe lack of Trp, resulting in the suppression of their proliferation. Meanwhile, metabolites such as pyridine carboxylic acid and kynurenine induce T cell apoptosis due to their obvious toxic effects. The suppression of effector T cells allows the tumor to escape host immune surveillance, thus creating an immunosuppressive microenvironment [25,26]. Furthermore, IDO acts on the dendritic cells (DCs) surface and interacts with regulatory T cells (Tregs), producing immunosuppressive effects due to their high expression, through the direct contact or production of suppressive cytokines. In turn, increased Tregs can bind to DC surface-associated receptors and induce IDO expression in DCs [27].

IDO is involved in immune regulation as an important negative immune regulator [28,29,30], and its regulatory mechanism mainly includes the following: (i) upon tryptophan depletion by IDO, the general control nonderepressible-2 (GCN2) kinase is activated, which then binds to tryptophan tRNA and inhibits the mammalian target of rapamycin (mTOR) kinase pathway to modulate immune responses; (ii) the AhR signaling pathway is regulated by kynurenine, the product catalyzed by IDO, resulting in the arrest of T cell activation, the differentiation of Treg cells, the alteration of DC functional immunogenicity, and other immunological responses; and (iii) IDO initiates downstream signaling effectors, including the noncanonical nuclear factor-κB (NF-κB) pathway, which result in sustained tumor growth factor (TGF)-β production and the downregulation of proinflammatory cytokine production in DCs, inducing type I interferons and plasmacytoid dendritic cell (pDC) phenotype. These results promote IDO1 expression, which in turn induces (TGF)-β secretion, thus creating a positive feedback loop. Noninflammatory DCs are generated by activating the noncanonical NF-κB pathway, thereby inhibiting T cell activation and promoting the development of Tregs [31]. In this context, these immunosuppressive effects allow IDO to play important roles in cancers, organ transplantations, and pathogenic infectious diseases [6,27,32,33,34,35,36].

## 3. Biochemical Properties of IDO in the KP

**Figure 3:**
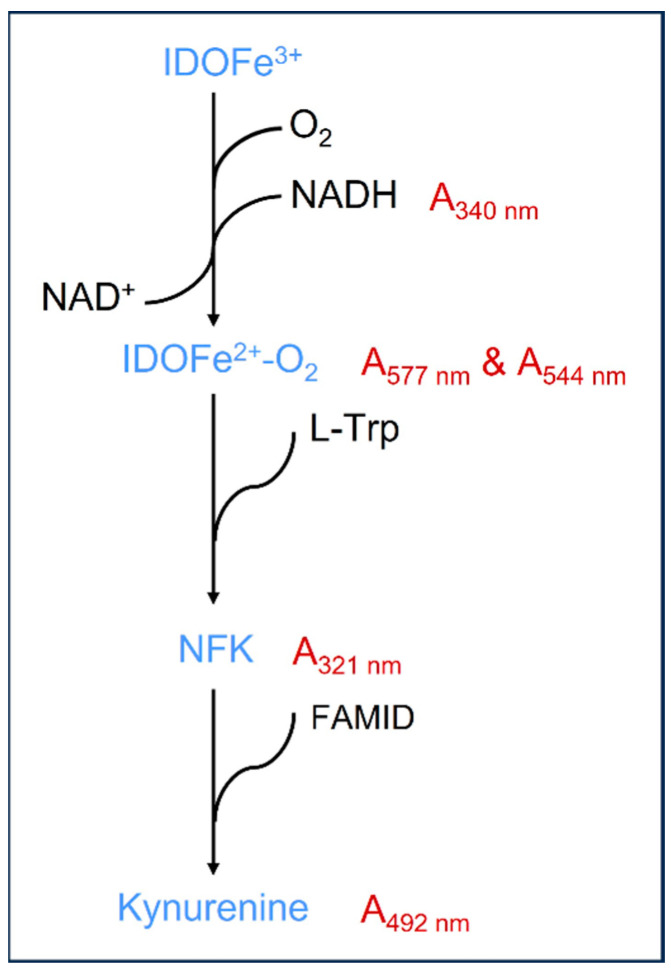
Strategy for the determination of indoleamine 2,3-dioxygenase (IDO) activity. IDOFe^3+^ can be converted to IDOFe^2+^-O_2_ in addition to β-NADH under aerobic conditions, resulting in increasing absorbance at 577 and 544 nm of the α and β transitions. The absorbance at 340 nm depicts the consumption of NADH. The incubation of NADH-generated IDOFe^2+^-O_2_ with L-Trp resulted in the production of NFK, as indicated by the increasing absorbance at 321 nm. NFK is rapidly converted to Kyn catalyzed by FAMID along with the increasing absorbance at 492 nm. NFK, N-Formyl kynurenine; FAMID, formamidase; Kyn, kynurenine.

Studies of IDO1 provided considerable insight into the properties of the enzyme, including substrate specificity, reactivity with dioxygen, and spectroscopic characteristics. Suzuki and co-workers reported that IDO1 was evolutionarily related to myoglobin with a molecular weight comparable with that of IDO1 [37,38,39]. An electron source is required to maintain IDO1 in the active ferrous form to overcome autoxidation to the ferric derivative [40,41]. Tryptophan catabolism begins with the break of the pyrrole ring by IDO1 to produce NFK. The cofactor of this enzyme is ferriporphyrin, and vitamin C is the activator of this enzyme, which has the function of protecting the Fe^2+^ in the cofactor from oxidation. It was found that IDO1 can oxidize NADH under aerobic conditions [42]. In experiments under aerobic conditions, IDOFe^3+^ was added to NADH in the absence of any other reactants, resulting in the formation of IDOFe^2+^-O_2_ with an increase in absorbance at both 577 nm and 544 nm (Figure 3). Subsequently, the reaction of IDOFe^2+^-O_2_ with L-Trp resulted in the production of NFK, as indicated by the increase in absorbance at 321 nm, with the generation of NAD^+^. A recent study demonstrated for the first time that an increase in immune-mediated IDO activity increases not only NAD^+^ biosynthesis but also NAD^+^ catabolism in primary human macrophages [43].

## 4. A Perspective Biomarker in a Variety of Human Diseases

Many studies have shown that IDO1 is closely associated with a variety of human diseases, including inflammatory diseases, microbial infections, autoimmunity, tumors and psychiatric disorders [27,44,45,46,47,48]. Trp depletion via IDO1 will disable the protein synthesis of T cells and inhibit the proliferation of T cells, resulting in the activation of GCN2 pathway and the suppression of mTOR pathway. Kyn will also activate AhR and then promote immune tolerance [32]. Immune response is the most common mechanism of most kidney diseases, the inhibition of which can delay the progress of kidney diseases. IDO activity was found to be associated with chronic kidney disease (CKD)-related indicators, such as calcium, phosphorus, uric acid, hemoglobin, albumin, coagulation indicators, and hypertension. Ischemic kidney injury can be protected by intervention in the IDO1/kynurenine pathway. In addition, increased IDO1 abundance and stress gene expression was shown in kidney tissue from patients with Ab-driven nephropathy. Therefore, IDO1 is supposed to be a promising biomarker to predict CKD and assess kidney function [49,50,51].

Recently, researchers used microarray data for the first time for immune cell infiltration analysis to identify IDO1 as a diagnostic and prognostic biomarker for diabetic nephropathy (DN) [52]. In addition, IDO expression is significantly induced by intrahepatic bacterial and viral infections as well as by non-pathogenic inflammatory conditions, such as liver fibrosis, cirrhosis, liver tumors, and certain liver parasites, which explains the role of IDO in liver-related diseases [53,54,55]. HBV and HCV infection can cause the upregulation of IDO and induce immunosuppression, while hepatitis B and C are closely associated with the development of hepatocellular carcinoma, so it can be further speculated that IDO may increase the risk of HBV and HCV-induced hepatocellular carcinoma [56,57]. Recent experiments showed that low IDO expression in asthmatic mice weakened immune tolerance, thus making it easier for bronchial asthma to develop or exacerbate [58].

On the one hand, IDO exerts immunosuppressive effects on the cellular microenvironment, leading to infections and immune escape of tumor cells; on the other hand, it also exerts suppressive effects on pathogens, such as bacteria and parasites, protecting the organism from pathogens to some extent. At present, it is found that IDO genes from parasites are related to the immunosuppressive effect of parasites and participate in the immune escape process of certain parasites, but the mechanism is still unclear [59,60]. Dai et al. confirmed that the activation of IDO gene in vivo was partially responsible for the anti-Toxoplasma effect mediated by IFN-γ [61]. In *Helicobacter pylori*-infected gastric mucosa, the activation of IFN-γ and the regulation of TGFB1 and CTLA4 together induced the upregulation of IDO1 expression [62]. This synergistic mechanism can promote immune tolerance and help bacteria evade host surveillance mechanisms and may reduce the effectiveness of bacterial eradication, promoting the progression of chronic gastritis. Therefore, for the above diseases involving abnormal IDO expression, the study of IDO inhibitors can be valuable in reversing the development and treatment of related diseases.

## 5. IDO Inhibitors in Clinical Trials for Cancer Immunotherapy

Thus, IDO has become an attractive target in cancer immunotherapy. A large number of IDO inhibitors have been reported, but most of the currently reported IDO inhibitors have still not reached clinical trials [63,64]. To date, six small molecule inhibitors of IDO1 have entered clinical studies, including indoximod (1-D-MT), epacadostat (INCB024360), BMS-986205, navoximod (GDC-0919, NLG-919), PF-06840003, and HTI-1090 (SHR9146) (Figure 1). The major clinical trials evaluating efficacy of these IDO inhibitors are shown in Table 1.

Indoximod (1-methyl-D-tryptophan, 1MT, NLG-8189) is the most representative tryptophan analogue inhibitor developed by the NewLink Genetics (NLNK) company [65]. Trp depletion can lead to the inhibition of mTORC1 signaling pathway. Indoximod, a Trp analogue, can reduce this inhibition and restore the activity of mTORC1, which is a central regulator for cell growth. Therefore, indoximod does not directly inhibit the enzymatic activity of IDO or TDO, but instead resists the effects elicited by these enzymes [66]. Indoximod has good oral bioavailability, safety, and tolerability in treating prostate cancer, breast cancer, non-small cell lung cancer, and brain tumors. It has been shown that the antitumor efficacy of indoximod is significantly enhanced when used in combination with other therapies [67,68].

Epacadostat (INCB024360), a competitive inhibition of IDO, effectively interferes with tryptophan metabolism by competing with tryptophan to bind to the catalytic domain of IDO [69]. Studies showed that epacadostat could reduce tumor growth and promote the proliferation of T cells and NK cells [70]. It is mainly used in ovarian cancer, melanoma, myelodysplastic syndrome, and myelodysplastic syndromes with a high specificity inhibition of IDO1 [71,72]. Moreover, HTI-1090 (SHR9146) is a novel and efficient small molecule IDO1/TDO dual inhibitor with a similar structure to epacadostat and has good oral bioavailability and safety. A Phase I trial of HTRI-1090 in advanced solid tumors was completed in January 2019.

Inrodostat mesylate (BMS-986205), a potent and selective oral IDO1 inhibitor, can specifically bind to IDO1 but not IDO2 or TDO [73]. BMS-986205 occupies the heme cofactor binding site to prevent the further activation of IDO1, thereby reversing the immunosuppression system in cancer patients, potentially improving cancer prognosis, especially when used in combination with other immunotherapies. BMS-986205 combined with Nivolumab in the treatment of liver cancer, melanoma, and solid tumors has been shown to be well tolerated in patients [74,75]. Navoximod (GDC-0919, NLG-919) is an orally active IDO1 inhibitor, contains the 4-phenylimidazole structure of tryptophan IDO inhibitor, which has stronger anti-IDO activity than epacadostat and BMS-986205. Studies showed that navoximod was able to restore the T cell function in vitro, and Kyn levels decreased after navoximod treatment in vivo. It is a promising novel immune checkpoint inhibitor for the treatment of recurrent solid tumors [76,77,78]. PF-06840003 is a non-competitive, non-hemoglobin-binding IDO inhibitor with high bioavailability, which was reported to restore the function of T cells in vitro and to reduce the Kyn levels in vivo. It can cross the blood–brain barrier and, therefore, may interfere with and inhibit tumor brain metastasis for the treatment of glioblastoma [79,80].

## 6. Applications of IDO Activity Assays

IDO activity induced by cellular immune activation plays a crucial role in tumor-promoting inflammatory processes, cancer formation and immunosuppressive therapy [81]. Recent studies have reported that IDO activity, as reflected by Kyn/Trp ratio, shows significant differences in different types of mental disorders. However, the results of studies on Kyn/Trp calculations for severe mental disorders are inconsistent [82]. Therefore, with the important involvement of IDO in various vital metabolic activities, it is necessary to summarize the currently available methods for the determination of IDO activity, hoping to find a suitable method for the determination of IDO activity to be applied in clinical diagnosis and treatment.

### 6.1. IDO Enzyme Activity Assays Based on Different Intermediates

In the kynurenine pathway, the formation of certain intermediates is accompanied by the changes of absorption value at different nanometer wavelengths. Therefore, we here briefly summarized the determination of different intermediates. First of all, IDO activity can be determined through the concentration of N-Formyl kynurenine, which is the first intermediate where tryptophan is catalyzed. The assay of DO1 enzyme activity was performed under normal atmospheric oxygen partial pressure (approximately 250 μM O_2_ in buffer at 24 °C) based on the method described by Shimizu et al. [83]. The experiment mixture contained potassium phosphate buffer, methylene blue, ascorbic acid, catalase, L-tryptophan, and the determined IDO1 enzyme [84]. The increase in absorbance at 321 nm due to the formation of N-Formyl kynurenine was continuously observed and recorded at 24 °C. IDO activity was then calculated according to the change of absorbance value in unit time (Figure 3).

Likewise, IDO activity can also be determined through the concentration of kynurenine produced. For example, the IDO activity can be determined in the cell homogenate via a slight modification of the method (Hara et al.) [85]. Briefly, 30% trichloroacetic acid (TCA) was added to the culture supernatant, vortexed, and spun. The supernatant was then added to an equal amount of Ehrlich reagent (100 mg/5 mL glacial acetic acid). The absorbance at 492 nm was recorded in multifunctional microplate reader (Figure 3). The change in kynurenine concentration was obtained by subtracting the kynurenine content of fresh uncultured media from the value derived from the cultured supernatant [43]. Currently, the ratio of kynurenine/tryptophan is also considered to determine the enzymatic activity of IDO [86]. In addition, the Kyn in the supernatant can also be analyzed by high performance liquid chromatography (HPLC).

Moreover, recent studies have shown that IDO1 can oxidize extra NADH to NAD^+^ under aerobic conditions. Through binding to NADH, IDO1 was confirmed in the oxidation of L-Trp with the conversion of IDOFe^3+^ to IDOFe^2+^-O_2_ (Figure 3). The NADH-supported oxidation of the L-Trp assay can be detected at three different absorption values as follows. First, the solutions of IDO1 and NADH were mixed in MOPS buffer and incubated at 20 °C. When the spectrum of the sample was completely converted to that of IDOFe^2+^-O_2_, the sample was mixed with an equal volume of L-Trp solution with the increasing absorption values at 577 nm and 544 nm that are characteristic of the α and β transitions in the spectrum of IDOFe^2+^-O_2_ [41]. Second, the reaction of the NADH-generated IDOFe^2+^-O_2_ with L-Trp resulted in the formation of NFK, as indicated by the increasing absorbance at 321 nm. Finally, the consumption of NADH could be detected in the decreasing absorbance maxima at 340 nm with the formation of NAD^+^ [42]. Moreover, the IDO activity in vivo can also be determined by NAD^+^, which is the end product of KP metabolism. The IFN-γ activation of mononuclear phagocytes significantly increases IDO and flux through the KP. It was first shown that an immune-mediated increase in IDO activity increased NAD^+^ production in IFN-γ-stimulated human primary mononuclear cells. Therefore, the determination of NAD^+^ is able to reflect the activity of IDO in vivo. The cellular pyridine nucleotide content (NAD^+^+NADH) can be measured by the Thiazolyl blue microcycling assay of Bernofsky and Swan adapted to a 96-well plate format [43].

### 6.2. IDO Activity Assays in Inhibitors In Vitro

In recent years, since IDO inhibitors have been effective in the treatment of a variety of tumors, the establishment of a stable IDO activity assay system is essential for the screening of novel and efficient IDO inhibitors and the evaluation of the effects of IDO mechanisms. The screening process for IDO inhibitors is generally as follows. The purified recombinant human IDO was constructed by genetic engineering. Taking a compound as the object, IDO activity can be initially determined by the several methods described above to obtain the inhibition type, half-inhibition concentration, and inhibition constant. Next, a high expression-human IDO plasmid was constructed and transfected with HEK 293 cells to evaluate the IDO inhibitory activity of the compound at the cellular level. MTT colorimetric assay was used to investigate the growth inhibitory effect of the compound on a specific tumor cell. Then, the animal experiments were performed to be verified by further studies. In addition, the yeast-based screening assay was also regarded as a powerful tool for the early stages of the identification of inhibitors of IDO [87]. Judging from these assays, the activity assay of IDO is crucial and central in the screening and evaluation of IDO inhibitors in vitro.

### 6.3. IDO Activity Assays in Human Plasma/Serum

Since IDO is a potential biomarker for many solid tumors, the detection of IDO activity in human blood is a direct indicator of the efficacy of tumor therapy. Currently, laboratories can detect the transcript level and expression level of IDO in serum via real-time PCR and Western blotting methods, respectively. Alternatively, the expression of IDO in cancer and paraneoplastic tissues can be determined through semi-quantitative immunohistochemistry. For example, HPLC is used to determine the concentration of Trp and Kyn in human plasma/serum, such as assessing the activity of IDO before and after treatment in patients with early stage NSCLC via Kyn and the kynurenine to tryptophan ratio (Kyn/Trp) in human blood [88]. This ratio is often used instead of the measurement of IDO activity. However, IDO is not the only determinant of the increase in the plasma (Kyn/Trp) ratio in vivo. This ratio is not accurate in vivo due to various factors such as the availability of nutrients, the presence of multiple cell types, complex structural and functional tissue arrangements, and the interactions of extracellular matrix, hormones, cytokines, and paracrine [86]. Moreover, HPLC has not been used clinically due to the inconvenient experiment and the high price. Therefore, the universal simple assay for direct detection of IDO activity in clinical tests is urgently needed.

## 7. Conclusions

IDO is an evolutionarily ancient enzyme that catalyzes the amino acid tryptophan metabolism, which is also an endogenous mechanism of immune-regulation and tolerance in the immune system. It suppresses the proliferation and differentiation of effector T cells, and significantly enhances the inhibitory activity of Tregs. Thus, this immune escape causes the immune system to fail to respond effectively to tumor antigens, which induces abnormal levels of IDO in host cells [64,89]. Based on these properties, IDO has become both a promising therapeutic target and an unfavorable prognostic biomarker with clinical potential for the diagnosis and treatment of tumor diseases [90,91]. At present, a number of clinical trials are actively underway that some chemotherapy drugs or vaccines against IDO to potentiate anti-Tregs can be combined with IDO inhibitor drugs in some cases to treat solid malignant tumors [92,93]. The current status of the study shows that this combined immunotherapy has shown positive therapeutic effects during the window of opportunity for conventional treatment of multiple tumor diseases [94].

As a consequence, the determination of IDO activity is particularly important both in the diagnosis of oncological diseases and in the evaluation of the effect of IDO inhibitors in immunotherapy. In addition to the assessment of the efficacy of solid malignant tumor therapy, the determination of IDO activity can also be applied to the diagnosis and therapeutic monitoring of various diseases, such as kidney disease, intestinal disease, cardiovascular disease, reproductive system, parasitic infections, mental illness, etc. At present, the determination of IDO activity in human plasma/serum has great application prospects in the diagnosis and treatment of a variety of tumor-related diseases. The calculation method of the Kyn/Trp ratio, which is widely used in experimental studies, cannot be applied to clinical practice because of its high price, inconvenience, and susceptibility to many other factors in vivo [86]. Therefore, the current experimental methods for the determination of IDO activity were summarized in this review. We expect that, based on these activity assays, a method for the direct determination of IDO activity will be available for clinical use in the near future.

## Figures and Tables

**Table 1:** IDO inhibitors in clinical trials.

Inhibitor	Phase	Medication	Tumor Type	ID	Status
Indoximod (1-D-MT)	I	Combination with idarubicin and cytarabine	Acute myeloid leukemia	NCT02835729	2019-12 completed
	II	Combination with docetaxel or paclitaxel	Metastatic breast cancer	NCT01792050	2017-07 completed
	I/II	Combination with ipilimumab, pembrolizumab and nivolumab	Metastatic melanoma	NCT02073123	2019-07 completed
	II	Combination with sipuleucel-T	Metastatic prostate cancer	NCT01560923	2018-12 completed
	I	Combination with ibrutinib, cyclophosphamide and etoposide	Pediatric brain cancer	NCT05106296	Recruiting
	I/II	Combination with temozolomide and bevacizumab	Newly diagnosed glioblastoma	NCT02052648	2019-06 completed
	I	Single agent	Advanced malignancy	NCT00567931	2012-09 completed
	I	Combination with temozolomide	Pediatric brain tumor	NCT02502708	2020-02 completed
	I/II	Combination with p53 DC vaccine	Metastatic breast cancer	NCT01042535	2018-02 completed
	I	Combination with docetaxel	Metastatic solid tumor	NCT01191216	2013-08 completed
	II	Combination with partial radiation	Progressive brain cancer	NCT04049669	Recruiting
Epacadostat (INCB024360)	II	Combination with pembrolizumab	Gastrointestinal stromal tumor	NCT03291054	2020-08 completed
	I	Combination with sirolimus	Advanced malignancy	NCT03217669	Active, not recruiting
	II	Combination with pembrolizumab	Gastroesophageal junction or gastric cancer	NCT03196232	2018-05 completed
	II	Single agent	Myelodysplastic syndrome	NCT01822691	2015-02 completed
	II	Combination with pembrolizumab	Locally advanced/metastatic sarcoma	NCT03414229	Active, not recruiting
	II	Combination with pembrolizumab	ovarian clear cell carcinoma	NCT03602586	Active, not recruiting
	I	Single agent	Advanced malignancy	NCT01195311	2013-07 completed
	I/II	Combination with durvalumab	Advanced solid tumor	NCT02318277	2020-10 completed
	III	Combination with pembrolizumab	Cisplatin-ineligible urothelial carcinoma	NCT03361865	2020-08 completed
	II	Combination with pembrolizumab	Metastatic non-small cell lung cancer	NCT03322540	2020-11 completed
	III	Combination with pembrolizumab	Metastatic urothelial carcinoma	NCT03374488	2020-07 completed
	I/II	Combination with MK-3475	High colorectal cancer, Endometrial cancer, Head and neck cancer and Hepatocellular carcinoma	NCT02178722	2020-11 completed
	I/II	Combination with intralesional SD101	Advanced/refractory solid tumors and lymphoma	NCT03322384	2020-04 completed
	I/II	Combination with nivolumab	Advanced solid tumors and lymphomas; squamous cell carcinoma of head and neck and non-small cell lung cancer	NCT02327078	2020-06 completed
	II	Combination with MELITAC 12.1 peptide vaccine	Stage III-IV melanoma	NCT01961115	2017-05 completed
	III	Combination with pembrolizumab	Locally advanced/metastatic renal cell carcinoma	NCT03260894	Active, not recruiting
	III	Combination with pembrolizumab	Unresectable or metastatic melanoma	NCT02752074	2019-08 completed
	II	Combination with retifanlimab, INCAGN02385 and INCAGN02390	Neoadjuvant urothelial carcinoma	NCT04586244	Recruiting
	I/II	Single agent	Locally advanced rectal cancer	NCT03516708	Recruiting
	I/II	Combination with DPX-Survivac and cyclophosphamide	Recurrent ovarian cancer	NCT02785250	Active, not recruiting
	I	Single agent	Epithelial ovarian, fallopian tube, or primary peritoneal cancer	NCT02042430	Active, not recruiting
	II	Combination with pembrolizumab	Metastatic pancreas cancer	NCT03006302	Active, not recruiting
	II	Combination with pembrolizumab and platinum-based chemotherapy	Metastatic non-small cell lung cancer	NCT03322566	2020-10 completed
	II	Combination with retifanlimab and pemigatinib	Advanced or metastatic endometrial cancer	NCT04463771	Recruiting
	III	Combination with pembrolizumab and cetuximab	Recurrent or metastatic head and neck squamous cell carcinoma	NCT03358472	Active, not recruiting
BMS-986205	II	Combination with nivolumab	Endometrial cancer	NCT04106414	Active, not recruiting
	I/II	Combination with nivolumab	Liver cancer	NCT03695250	Active, not recruiting
	I/II	Combination with nivolumab	Advanced malignant solid tumor	NCT03792750	2020-12 completed
	III	Combination with nivolumab	Advanced melanoma	NCT03329846	2020-07 completed
	II	Combination with nivolumab	Squamous cell cancer of the head and neck	NCT03854032	Active, not recruiting
	I	Combination with nivolumab	Newly diagnosed glioblastoma	NCT04047706	Active, not recruiting
	I/II	Combination with nivolumab, relatlimab and ipilimumab	Advanced tumor	NCT03459222	Active, not recruiting
	II	Combination with nivolumab, relatlimab and ipilimumab	Advanced renal cell carcinoma	NCT02996110	2021-11 completed
	I	Combination with nivolumab	Advanced malignant tumor	NCT03192943	2018-12 completed
	I/II	Combination with nivolumab, and ipilimumab	Advanced malignant tumor	NCT02658890	2021-10 completed
	II	Combination with nivolumab, relatlimab and ipilimumab	Advanced gastric cancer	NCT02935634	2022-05 completed
	III	Combination with nivolumab, gemcitabine and cisplatin	Muscle invasive bladder cancer	NCT03661320	Active, not recruiting
	I	Combination with nivolumab, relatlimab and cabiralizumab	Solid tumor	NCT03335540	2021-08 completed
Navoximod (GDC-0919, NLG-919)	I	Single agent	Advanced solid tumor	NCT02048709	2016-02 completed
	I	Combination with atezolizumab	Locally advanced or metastatic solid tumors	NCT02471846	2019-10 completed
PF-06840003	I	Single agent	Malignant gliomas	NCT02764151	Terminated
HTI-1090 (SHR9146)	I	Single agent	Advanced solid tumor	NCT03208959	2019-01 completed
	I	Combination with SHR-1210 and apatinib	Advanced solid tumor	NCT03491631	Active, not recruiting

Data is available at ClinicalTrials.gov, https://www.clinicaltrials.gov.

## Data Availability

Data sharing is not applicable to this article as no datasets were generated or analyzed in the current study.
